# GRAMD1B regulates cell migration in breast cancer cells through JAK/STAT and Akt signaling

**DOI:** 10.1038/s41598-018-27864-6

**Published:** 2018-06-22

**Authors:** Puja Khanna, Joan Shuying Lee, Amornpun Sereemaspun, Haeryun Lee, Gyeong Hun Baeg

**Affiliations:** 1 Department of Anatomy, Yong Loo Lin School of Medicine, National University of Singapore, Singapore, MD10, 4 Medical Drive, 117594 Singapore; 2 Department of Anatomy, Faculty of Medicine, Chulalongkorn University, Bangkok, 10330 Thailand; 3 Department of Life Sciences, Pohang University of Science and Technology, Pohang, 37673 South Korea

## Abstract

Dysregulated JAK/STAT signaling has been implicated in breast cancer metastasis, which is associated with high relapse risks. However, mechanisms underlying JAK/STAT signaling-mediated breast tumorigenesis are poorly understood. Here, we showed that GRAMD1B expression is upregulated on IL-6 but downregulated upon treatment with the JAK2 inhibitor AG490 in the breast cancer MDA-MB-231 cells. Notably, *Gramd1b* knockdown caused morphological changes of the cells, characterized by the formation of membrane ruffling and protrusions, implicating its role in cell migration. Consistently, GRAMD1B inhibition significantly enhanced cell migration, with an increase in the levels of the Rho family of GTPases. We also found that *Gramd1b* knockdown-mediated pro-migratory phenotype is associated with JAK2/STAT3 and Akt activation, and that JAK2 or Akt inhibition efficiently suppresses the phenotype. Interestingly, AG490 dose-dependently increased p-Akt levels, and our epistasis analysis suggested that the effect of JAK/STAT inhibition on p-Akt is *via* the regulation of GRAMD1B expression. Taken together, our results suggest that GRAMD1B is a key signaling molecule that functions to inhibit cell migration in breast cancer by negating both JAK/STAT and Akt signaling, providing the foundation for its development as a novel biomarker in breast cancer.

## Introduction

Breast cancer is a clinically heterogeneous disease and has been ranked as the most common malignancy in women worldwide, with incidence rates particularly high in developed countries and relative mortality rates greatest in less developed countries^1,2^. Remarkably, breast cancer-associated mortality has been attributed to metastases of the cancer to secondary distant sites, rather than to the primary tumor^2^. 10–15% of breast cancer patients show symptoms of metastasis within 3 years of initial detection of tumor, while others may show symptoms after 10 years or more^3^. This heterogeneity in metastatic rates of breast tumors further adds to the complexity of the disease and makes prognosis, as well as the development of treatment strategies, difficult.

Metastatic breast tumors are the more chemoresistant forms of breast cancer and are hence associated with poor survival^4^. The JAK/STAT cascade, a cytokine and growth factor signaling pathway^5^, has been well established in breast tumorigenesis^6^. Constitutively-activated STAT3 has been detected in approximately 50–60% of all breast cancers^7^  where it contributes to several hallmarks of cancer, including proliferation, angiogenesis and metastasis^6^. In particular, the autocrine/paracrine IL-6/JAK/STAT3 feed-forward loop has been implicated as a key player of tumor progression and metastasis^8^. Immunohistochemical analyses with human breast tumor samples further revealed an increased level of IL-6 at the leading edge of invasive breast tumors, with its level positively correlated with advanced stage, confirming a pivotal role of IL-6 signaling in breast tumor metastasis *in vivo*^8^.

Another signaling pathway commonly dysregulated in breast cancer is the PI3K/Akt signaling cascade^9,10^. Mutations in genes of this signaling pathway have been reported in more than 70% of breast tumors^9^ where they were found to promote tumor growth and progression, and contribute to chemoresistance^11,12^. Furthermore, PI3K/Akt signaling was shown to promote the expression of Twist, a master transcription factor of epithelial-mesenchymal transition (EMT), leading to enhanced TGF-β receptor signaling which in turn functions to maintain hyperactivated PI3K/Akt signaling, cooperatively driving breast tumor metastasis^13,14^. More recently, cPLA2α was also reported to facilitate TGF-β-induced EMT in breast cancer through the PI3K/Akt signaling cascade^15^, suggesting the pivotal role of PI3K/Akt signaling in breast cancer cell migration. Interestingly, several studies have also reported a cross-talk between PI3K/Akt and JAK/STAT signaling in metastatic breast tumors^4,16^. However, there is a gap in knowledge of the exact molecular mechanisms by which these signaling cascades interact with each other to facilitate breast cancer metastasis.

A genome-wide RNAi screen was conducted in *Drosophila* to identify additional signaling components of the JAK/STAT pathway^17^. The *Drosophila* ortholog of GRAMD1B (GRAM domain-containing protein 1B) was identified as a putative component of the signaling cascade^17^. GRAMD1B contains a GRAM domain that is known to function as a protein-binding or lipid-binding intracellular signaling domain^18,19^. More recently, GRAMD1B has been implicated in human malignancies. Specifically, it was reported to play a role in chemoresistance of ovarian cancer patients, such that GRAMD1B inhibition led to an anti-tumor effect^20^. Furthermore, a genome-wide association study in chronic lymphocytic leukemia patients revealed that single nucleotide polymorphism in *Gramd1b* is associated with increased risk of disease in a European population^21^. In gastric cancer, GRAMD1B regulates cell survival by upregulating expression of the anti-apoptotic molecule Bcl-xL^22^. In this study, JAK/STAT signaling was found to positively regulate GRAMD1B expression in the breast cancer MDA-MB-231 cells. Knockdown of *Gramd1b* resulted in distinct morphological changes of the cells, accompanied by increased rates of cell migration. Intriguingly, p-JAK2 and p-Akt levels were drastically induced upon GRAMD1B inhibition, but treatment with AG490 or MK-2206 almost completely suppressed the pro-migratory phenotypes induced by *Gramd1b* knockdown. Lastly, our epistasis analysis suggested a central role of GRAMD1B in the linkage between JAK/STAT and Akt signaling.

## Results

### GRAMD1B expression is regulated by JAK/STAT signaling in the breast cancer MDA-MB-231 cells

**Figure 1:**
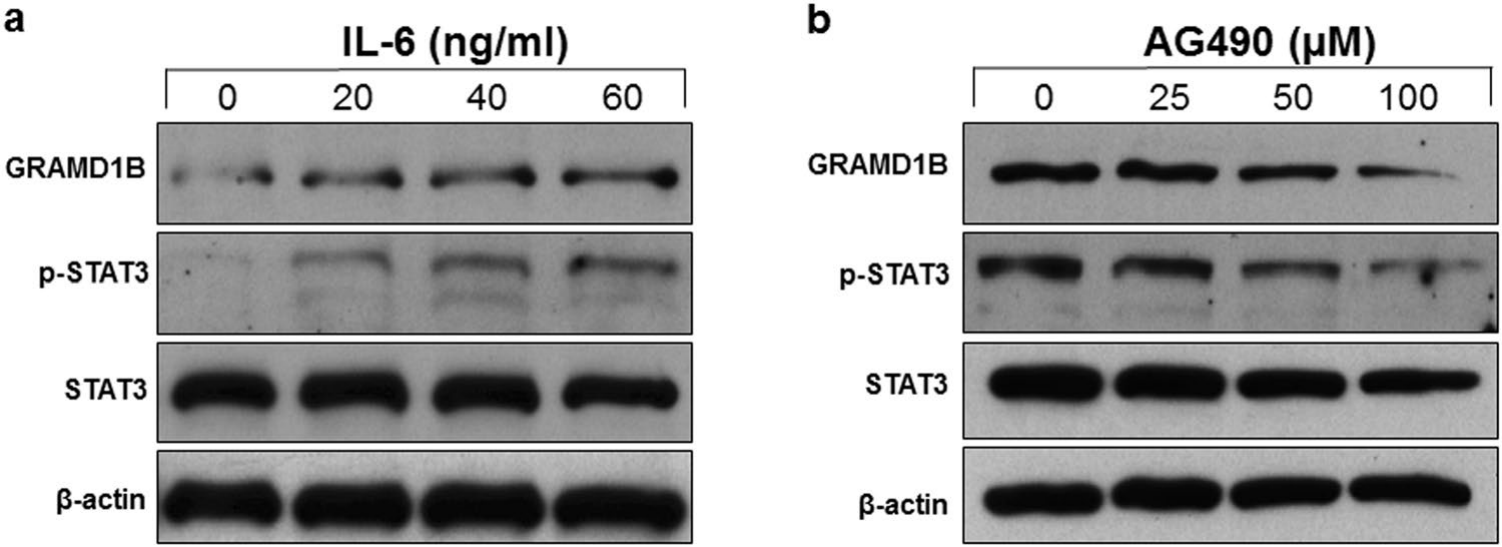
JAK/STAT signaling regulates GRAMD1B expression in the breast cancer MDA-MB-231 cells. (**a**) IL-6-induced JAK/STAT signaling increases GRAMD1B expression. Full-length blots are included in Supplementary Fig. S6. (**b**) Decrease in GRAMD1B expression is observed on AG490-mediated JAK2 inhibition. Full-length blots are included in Supplementary Fig. S7.

The JAK/STAT cascade has been shown to transcriptionally regulate its components such as the SOCS family of proteins, which in turn regulate JAK/STAT signaling activity, thus generating a feedback loop^23,24^. Since the *Drosophila* ortholog of GRAMD1B was initially identified as a signaling component of the *Drosophila* JAK/STAT pathway^17^, we determined whether GRAMD1B expression is also modulated by JAK/STAT signaling in the breast cancer MDA-MB-231 cells. Interestingly, we observed an increase in the expression of GRAMD1B of 49 kDa (UniProtKB Q3KR37-3) on IL-6 stimulation (Fig. 1a). By contrast, GRAMD1B expression was down-regulated by the JAK2 inhibitor AG490 (Fig. 1b), suggesting that the JAK/STAT cascade regulates GRAMD1B expression in breast cancer cells.

### GRAMD1B inhibition causes morphological changes of breast cancer cells

**Figure 2:**
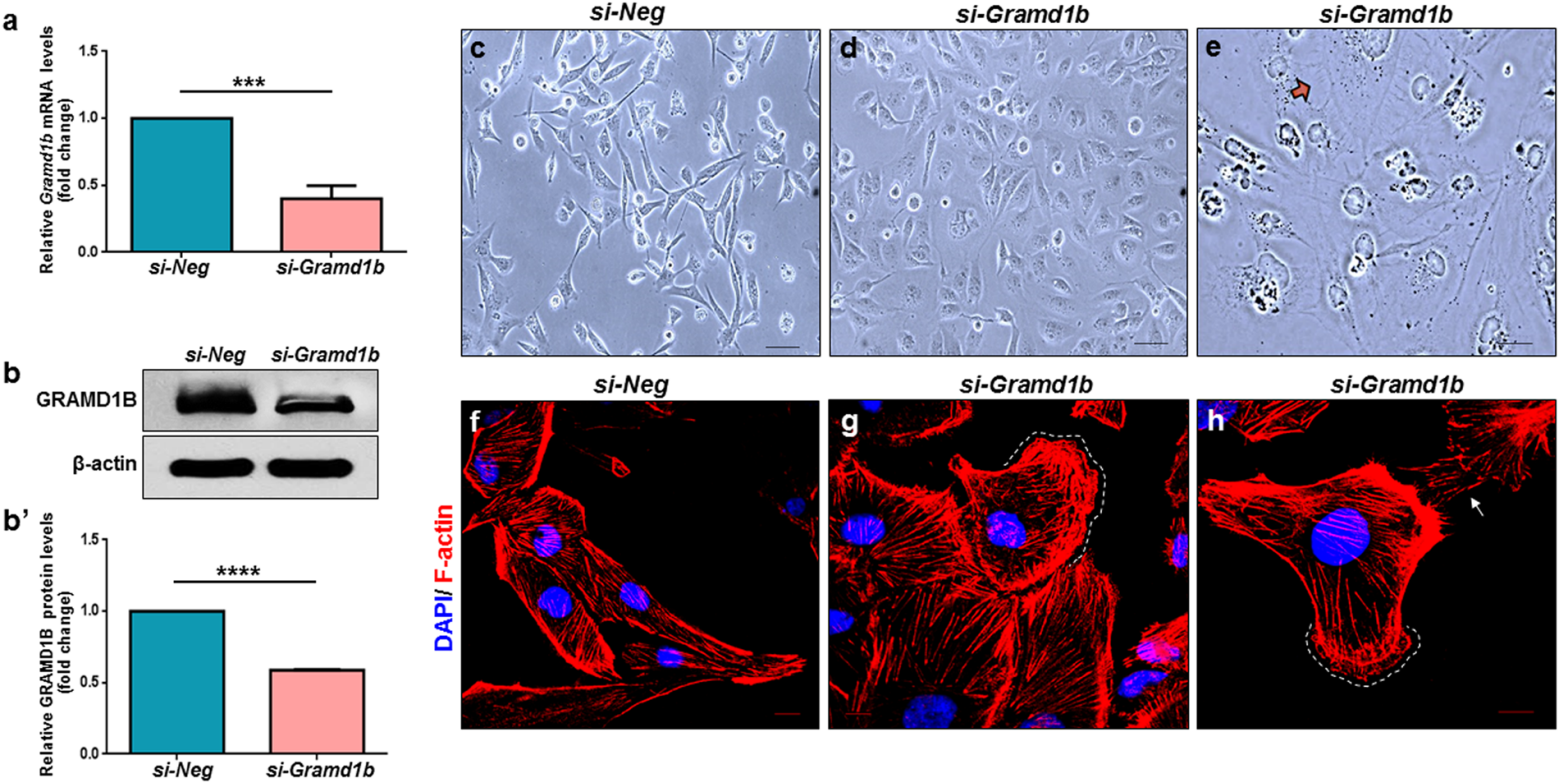
GRAMD1B inhibition causes morphological changes of breast cancer cells. (**a**) *si-Gramd1b* knockdown decreases *Gramd1b* mRNA levels. Data is represented as mean ± SEM of n = 4. (**b**-b’) A decrease in GRAMD1B protein levels is also observed in *Gramd1b* knockdown cells. Data is represented as mean ± SEM of n = 3. (**c**,**d**) *Gramd1b* knockdown transforms the parental spindle-shaped MDA-MB-231 cells into rounded and flattened cells. (Scale bar = 100 μm). (**e**) Note the presence of cell membrane protrusions on loss of GRAMD1B activity (arrow). (Scale bar = 50 μm). (**f**–**h**) Phalloidin staining of *si-Gramd1b* transfected cells reveals the presence of F-actin-rich membrane protrusions (arrow), accompanied by membrane ruffle formation (white dashed lines). (Scale bar = 10 μm). ***P < *0.001*; ****P < *0.0001*.

To examine the function of GRAMD1B in JAK/STAT signaling-mediated biological processes such as cell invasion and migration in breast cancer cells, we first assessed the potency of *si-Gramd1b*, and found that it can efficiently knockdown *Gramd1b*, as evidenced by the significant reduction of both mRNA (Fig. 2a) and protein levels (Fig. 2b,b’). Interestingly, we noticed that the parental MDA-MB-231 cells which exhibit a spindle-shaped morphology (Fig. 2c), become rounded and flattened in shape on *Gramd1b* knockdown, (Fig. 2d), with the occurrence of cell membrane protrusions (Fig. 2e, arrow). In support of this, phalloidin staining of the *Gramd1b* knockdown cells revealed the presence of F-actin-rich membrane protrusions (arrow) accompanied by membrane ruffling (dotted line) (Fig. 2f–h), hallmarks of cell motility^25^, suggesting the putative function of GRAMD1B in cell migration. To exclude possible off-target results of *si-Gramd1b*, we used another siRNA targeting *Gramd1b* (*si-Gramd1b-2*) and confirmed a change in morphology of MDA-MB-231 cells, with formation of membrane protrusions (Supplementary Figs S1 and S2).

### GRAMD1B inhibition promotes cell migration in breast cancer cells

**Figure 3:**
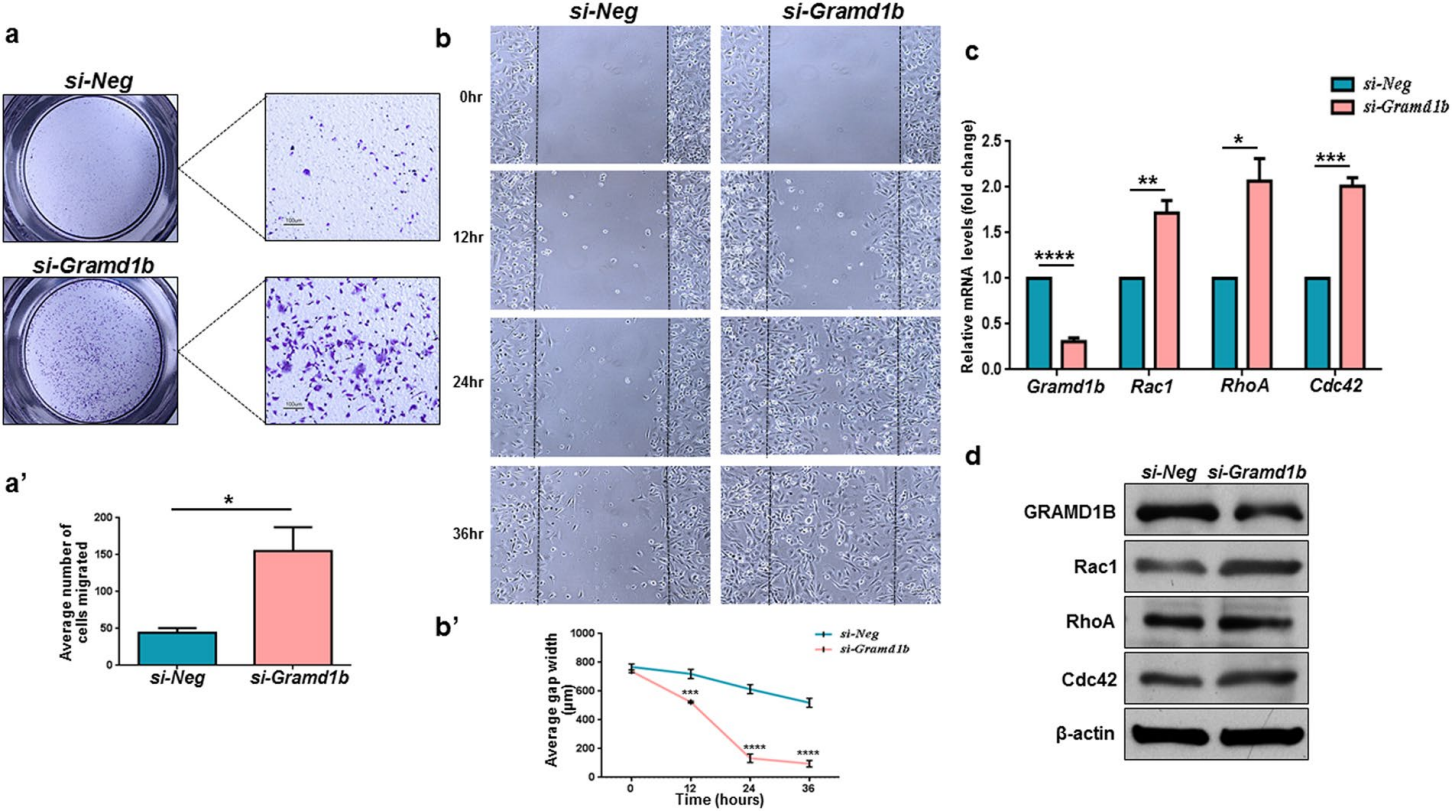
GRAMD1B inhibition promotes cell migration in breast cancer cells. (**a**-a’) Transwell migration assay shows an increase in cell migration on *Gramd1b* knockdown. (Scale bar = 100 μm). Data is represented as mean ± SEM of n = 3. (**b**-b’) Wound healing assay confirms the inhibitory effects of GRAMD1B on cell migration. Data is represented as mean ± SEM of n = 3. (**c**,**d**) qRT-PCR and Western blot analyses show a significant induction of the Rho GTPase Rac1, RhoA and Cdc42 at both mRNA and protein levels. Data is represented as mean ± SEM of n = 3. *P < *0.05*; **P < *0.01*; ***P < *0.001*; ****P < *0.0001*.

To further explore the potential role of GRAMD1B in cell migration, we conducted the transwell migration assay. Cells with reduced GRAMD1B activity showed significantly higher migration rates as compared to control cells (Fig. 3a,a’). This pro-migratory phenotype was further confirmed by the wound healing assay. While *Gramd1b* knockdown cells almost entirely covered the wound by 36 hours, only ~40% of the wound had been covered by control cells (Fig. 3b,b’), suggesting that GRAMD1B negatively regulates cell migration in breast cancer cells. Since the Rho family of GTPases have been implicated in modulating the dynamics of actin cytoskeleton, thereby controlling directional migration^26^, we next assessed whether GRAMD1B can modulate their expression. Interestingly, we detected an increase in both mRNA and protein levels of Rac1, RhoA and Cdc42 upon *Gramd1b* knockdown (Fig. 3c,d). Furthermore, inhibitors targeting the members of the Rho family of GTPases efficiently suppressed changes in cell morphology observed upon *Gramd1b* knockdown (Supplementary Fig. S3). These findings suggest that GRAMD1B inhibition-mediated morphological changes of the cells, followed by increased migratory rates, may be associated with the Rho family of GTPases.

### GRAMD1B negates JAK/STAT signaling

**Figure 4:**
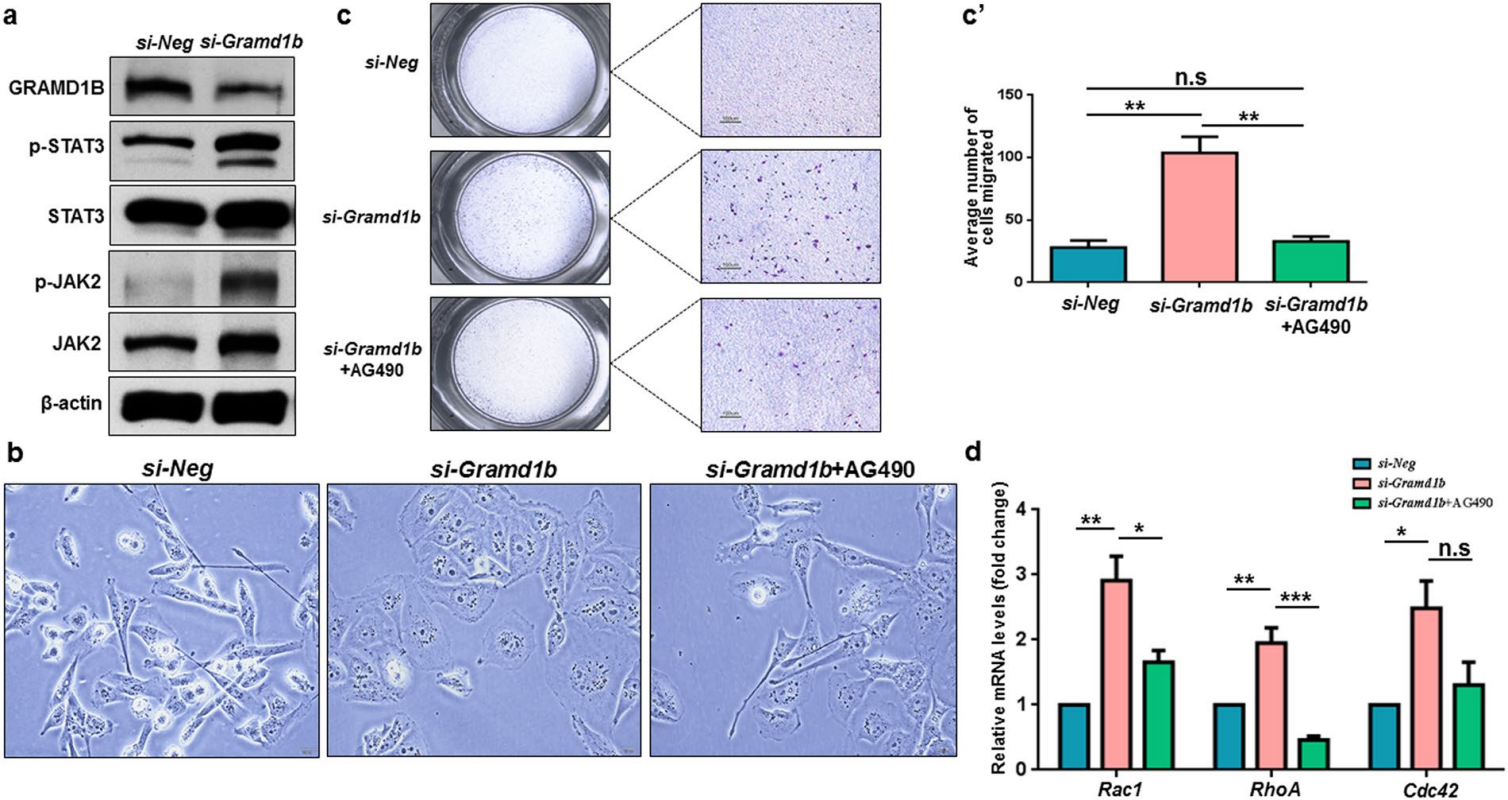
GRAMD1B negates JAK/STAT signaling. (**a**) Effects of GRAMD1B inhibition on JAK/STAT signaling. Increased p-JAK2 and p-STAT3 levels are detected on *Gramd1b* knockdown, suggesting that GRAMD1B negatively regulates JAK/STAT signaling. Full-length blots are included in Supplementary Fig. S8. (**b**) Treatment with AG490 almost completely suppresses the morphological changes of cells induced by *Gramd1b* knockdown. (Scale bar = 20 μm). (**c**-c’) Transwell migration assay reveals that AG490 co-treatment efficiently suppresses the enhanced cell migration caused by GRAMD1B inhibition. (Scale bar = 100 μm). Data is represented as mean ± SEM of n = 3. (**d**) qRT-PCR analysis shows that AG490 treatment efficiently blocks the induction of *Rac1* and *RhoA* in cells transfected with *si-Gramd1b*. Data is represented as mean ± SEM of n = 3. *P < *0.05*; **P < *0.01*; ***P < *0.001*.

The IL-6/JAK/STAT3 feedback loop has been well documented in breast tumor growth and metastasis^27^. Notably, increased expression levels of IL-6 and p-STAT3 have been detected at the leading edge of breast tumors and linked to advanced disease, suggesting a mechanistic role of the JAK/STAT cascade in promoting breast tumor progression^8,28^. Since GRAMD1B is positively regulated by the JAK/STAT pathway, and its inhibition facilities cell migration, we hypothesized that cell migration facilitated by the loss of GRAMD1B activity might be a result of JAK/STAT signaling activation, as some of STAT transcriptional targets such as SOCS negatively feedback to suppress JAK/STAT signaling^23,24^. Interestingly, we observed a dramatic induction of p-JAK2 and its downstream target p-STAT3 upon *Gramd1b* knockdown (Fig. 4a). This suggests that the JAK/STAT transcriptional target GRAMD1B negates the signaling by regulating JAK2 activity and that the increased migratory rates observed in *Gramd1b* knockdown cells are due to JAK/STAT signaling activation. To verify this hypothesis, we then examined the effects of GRAMD1B inhibition on cell morphology and migration in the presence of the JAK2 inhibitor AG490. We observed that JAK/STAT signaling inhibition almost completely reversed the flattened cells back into their original spindle-shaped morphology (Fig. 4b) and significantly suppressed the enhanced cell migration (Fig. 4c,c’). Furthermore, our qRT-PCR analysis showed that AG490 treatment efficiently blocked the increase in the expression of *Rac1* and *RhoA* induced by *Gramd1b* knockdown (Fig. 4d).

### GRAMD1B inhibition increases Akt signaling

**Figure 5:**
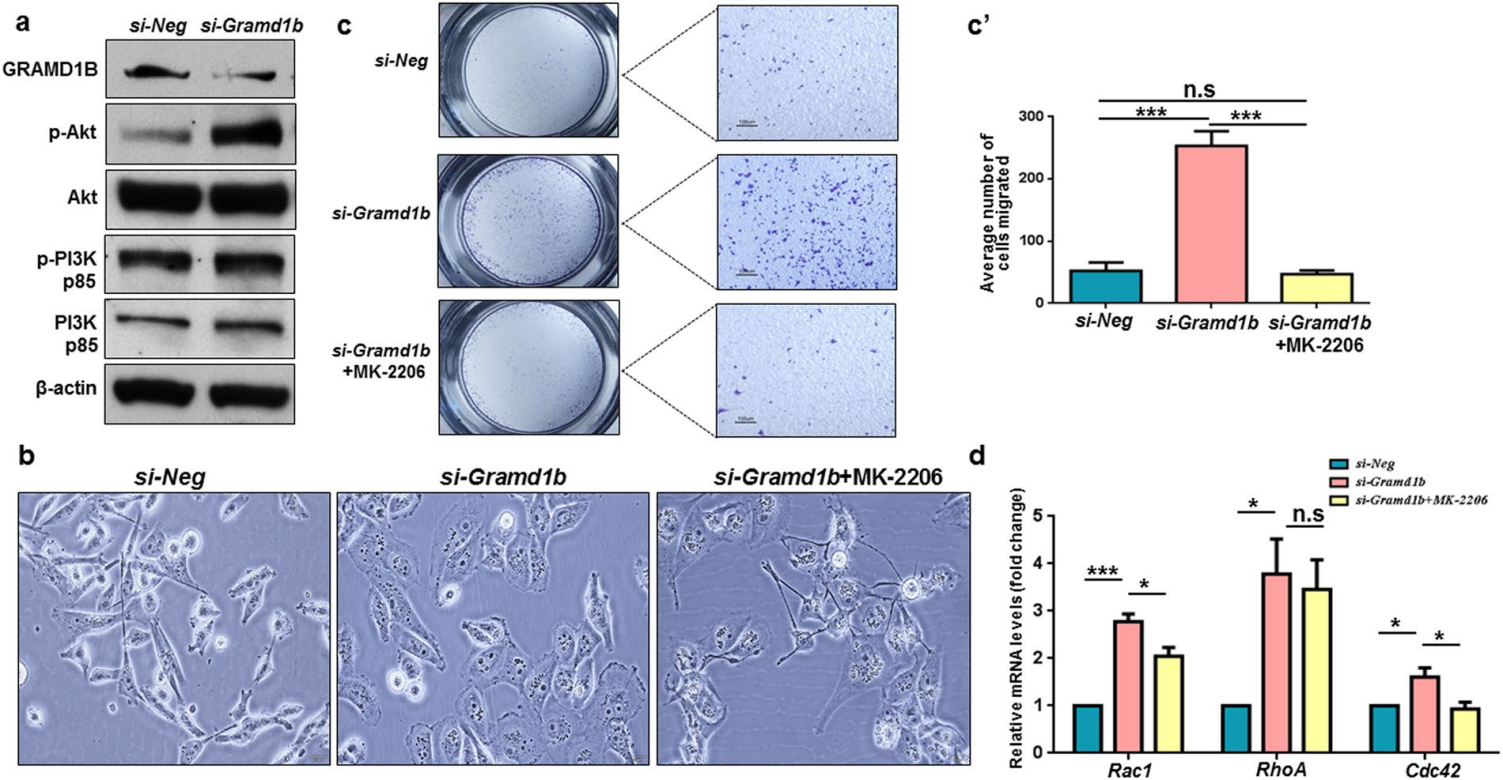
GRAMD1B inhibition increases Akt signaling. (**a**) Inhibitory effects of GRAMD1B on PI3K/Akt signaling. *Gramd1b* knockdown results in a dramatic increase in p-Akt levels but fails to alter p-PI3K levels, suggesting that GRAMD1B acts downstream to PI3K but upstream/parallel to Akt. Full-length blots are included in Supplementary Fig. S9. (**b**) The morphology changes of *Gramd1b* knockdown cells are negated in the presence of the pan-Akt inhibitor MK-2206. (Scale bar = 20 μm). (**c**-c’) MK-2206 treatment suppresses the GRAMD1B inhibition-enhanced cell migration. (Scale bar = 100 μm). Data is represented as mean ± SEM of n = 3. (**d**) Treatment of cells with MK-2206 significantly inhibits the expression of *Rac1* and *Cdc42* induced by *Gramd1b* knockdown. Data is represented as mean ± SEM of n = 3. *P < *0.05*; ***P < *0.001*.

The PI3K/Akt signaling pathway has been found to be frequently dysregulated in breast tumors where it promotes cell migration and invasion, as well as contributes to chemoresistance^9–11^. Since GRAMD1B inhibition facilitates cell migration, we asked whether it interacts with the PI3K/Akt pathway in cell migration. We first assessed the inhibitory effects of GRAMD1B on PI3K and Akt activation, and found that cells transfected with *si-Gramd1b* showed no change in p-PI3K levels but exhibited a significant increase in p-Akt, whereas total PI3K and Akt levels remained unchanged (Fig. 5a). This finding suggests that GRAMD1B inhibition-enhanced cell migration is associated with Akt activation and that inhibition of Akt may block the enhanced cell migration. Interestingly, the effects of GRAMD1B inhibition on cell morphology (Fig. 5b) and migration (Fig. 5c,c’) were efficiently suppressed in the presence of MK-2206, an oral pan-Akt inhibitor. Furthermore, co-treatment of cells with *si-Gramd1b* and MK-2206 significantly inhibited the expression of *Rac1* and *Cdc42* induced by *Gramd1b* knockdown (Fig. 5d). These findings suggest that GRAMD1B may act downstream to PI3K, but upstream or parallel to Akt in the regulation of cell migration.

### GRAMD1B plays a key role in the linkage between JAK/STAT and Akt signaling

**Figure 6:**
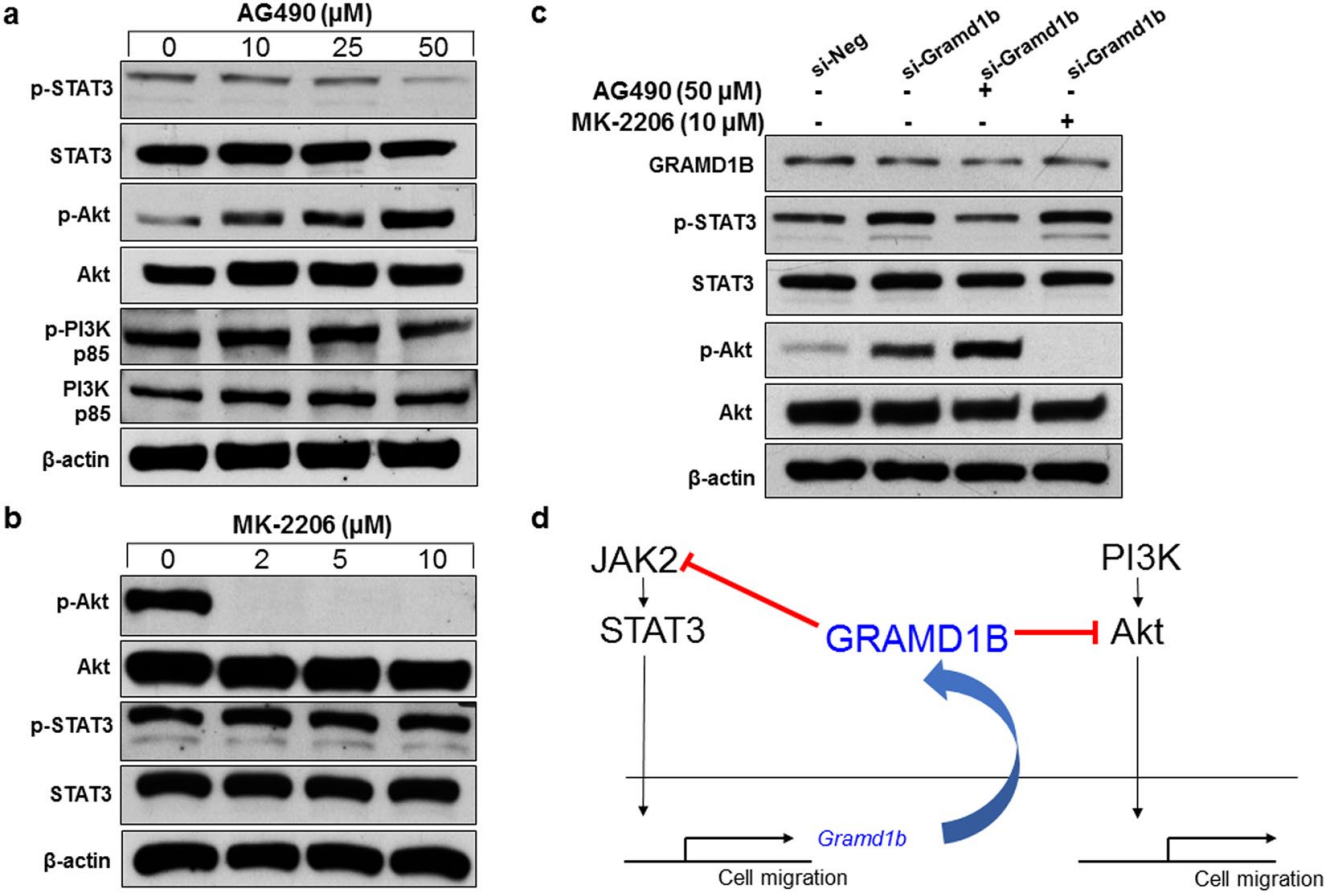
GRAMD1B plays a key role in the linkage between JAK/STAT and Akt signaling. (**a**) Effects of JAK/STAT signaling inhibition by AG490 on PI3K/Akt signaling. Note that AG490 dose-dependently increases p-Akt levels but does not affect p-PI3K levels, suggesting that JAK/STAT signaling acts downstream to PI3K but upstream/parallel to Akt. Full-length blots are included in Supplementary Fig. S10. (**b**) Effects of Akt signaling inhibition on JAK/STAT signaling. Note that p-STAT3 levels are not altered by MK-2206. Full-length blots are included in Supplementary Fig. S11. (**c**) GRAMD1B inhibition-induced p-Akt levels are further increased in the presence of AG490. Full-length blots are included in Supplementary Fig. S12. (**d**) Schematic for the mechanism of action of GRAMD1B, suggesting its pivotal role in the regulation of JAK/STAT and Akt signaling.

Since GRAMD1B is involved in the regulation of both JAK/STAT and Akt signaling, we next asked whether JAK/STAT and Akt signaling mutually regulate each other. Inhibitory effect of the JAK/STAT cascade on PI3K/Akt signaling was examined by treating cells with increasing concentrations of AG490. As expected, AG490 decreased p-STAT3 levels. Interestingly, AG490 treatment resulted in a dose-dependent increase in p-Akt levels, whereas it failed to affect p-PI3K levels (Fig. 6a). This finding suggests that JAK/STAT signaling acts downstream to PI3K, but upstream or parallel to Akt which is similar to the mechanism of action of GRAMD1B on PI3K/Akt signaling. On the other hand, inhibition of Akt signaling by MK-2206 did not cause any drastic alteration in p-STAT3 levels (Fig. 6b). To understand the molecular mechanism underlying the increase in p-Akt levels by JAK/STAT signaling inhibition, we performed epistasis analysis in cells treated with *si-Gramd1b* in the presence of AG490 or MK-2206. As expected, knockdown of *Gramd1b* alone caused an increase in both p-STAT3 and p-Akt levels. The increased p-STAT3 levels were diminished by AG490 treatment, but were not altered in the presence of MK-2206. However, interestingly, the elevated p-Akt levels by GRAMD1B inhibition were further enhanced in the presence of AG490 but completed suppressed by MK-2206 (Fig. 6c). Considering the fact that both JAK/STAT signaling and GRAMD1B act upstream or parallel to Akt, these findings may suggest that inhibitory effects of JAK/STAT signaling on Akt activation is through the regulation of GRAMD1B expression (Fig. 6d).

## Discussion

The JAK/STAT pathway functions as a key regulator of a wide variety of physiological and biological processes^5,29,30^. Thus, dysregulated signaling is often associated with various pathological conditions, including cancer. In breast tumors, persistently-active STAT3 has been found to promote breast tumor progression by facilitating cancer cell proliferation, angiogenesis and EMT^6^. Particularly, the IL-6/JAK/STAT3 autocrine activation loop is a key driver of cancer progression and metastasis in breast cancer^8,27^. However, there is a gap in our knowledge of the downstream effectors and signaling mechanisms underlying JAK/STAT-mediated breast carcinogenesis. Here, we showed that JAK/STAT signaling positively regulates GRAMD1B expression, which in turn negates the signaling in breast cancer cells, signifying the existence of a negative feedback mechanism. Moreover, we provided evidence that GRAMD1B modulates breast cancer cell migration through the regulation of both JAK/STAT and Akt signaling. Lastly, our epistasis analysis suggested the pivotal function of GRAMD1B in mediating the inhibitory effect of JAK/STAT signaling on Akt activity in breast cancer cells.

Intriguingly, loss of GRAMD1B activity transformed the parental spindle-shaped MDA-MB-231 cells into rounded and flattened cells (Fig. 2c–e), which are often associated with extensive cell migration^31^. During the process of metastasis, cancer cells also undergo transformation into rounded and flattened cells, conferring a cell migratory advantage to metastasize from the primary tumor to secondary sites^32^. Furthermore, *si*-*Gramd1b* knockdown cells exhibited F-actin-rich protrusions at the cell leading edges, as well as membrane ruffle formation (Fig. 2f–h). F-actin-rich membrane cell protrusions at the leading edge of motile cells are known to serve as one of the key driving forces in cell migration and extension^25^. In addition to this, membrane ruffling which often precedes the formation of lamellipodia is essential among other events for cell motility^33^. These observations led us to postulate that the GRAMD1B-associated cell morphology changes may play an important role in breast cancer cell migration. As expected, we observed a dramatic increase in cell migration rates (Fig. 3a,b) on *Gramd1b* knockdown, and detected increased expression of Rac1, RhoA and Cdc42 (Fig. 3c,d), which play a central role in the regulation of the actin cytoskeleton and cellular migration^34–36^. Increased Rac1 activity promotes ruffling and stimulates actin polymerization to generate lamellipodia through the activation of the ARP2/3 complex^26,37^. On the other hand, RhoA activation is required for the formation of stress fibres, which function in cellular contractility that is essential for driving cell migration^38^, and Cdc42 is responsible for the formation of filopodia that is often found to be active at the front of migrating cells, resulting in increased directional migration^39^. Hence, it is conceivable that GRAMD1B functions to suppress cell migration by regulating the expression of the Rho family of GTPases. Nonetheless, further analyses are required to determine the mechanism underlying the interaction between GRAMD1B and Rho GTPases in modulating breast cancer cell migration.

The JAK/STAT signaling pathway is one of many control pathways that promotes cell motility by regulating actin dynamics and activating key metastasis-promoting genes^40^. In particular, STAT3 activation induced by interleukin family of cytokines can promote migration and invasion *via* the regulation of downstream target molecules such as Vimentin,  Twist, MMP-9 and MMP-7^6^. Moreover, JAK2 activation mediates WASF3 upregulation, which subsequently promotes the formation of lamellipodia and increases cell migration *via* recruitment of ARP2/3^41^. We showed that IL-6-induced JAK/STAT activation increases GRAMD1B expression, whereas blockage of JAK2 activity decreases its expression (Fig. 1a,b). Notably, inhibition of GRAMD1B resulted in the induction of JAK2 activity (Fig. 4a), suggesting that a negative feedback mechanism exists. Several STAT downstream target genes are known to feedback into the circuitry and affect activity^24^. For instance, cytokine stimulation induces the production of SOCS proteins, which in turn negate JAK/STAT signaling by either directly inactivating JAK, blocking access of the STAT molecules to receptor binding sites, or promoting ubiquitination of JAK and/or STAT^23,42^. Considering the essential roles of JAK/STAT signaling in cell migration, our study suggests that GRAMD1B inhibition-enhanced cell migration is mediated by the activation of JAK/STAT signaling. In support of this, we detected that treatment of *Gramd1b* knockdown cells with AG490 almost completely bypassed the inhibitory effects of GRAMD1B on cell morphology (Fig. 4b) and migration (Fig. 4c,c’), as well as blocked the increase in the expression of *Rac1* and *RhoA* induced by *Gramd1b* knockdown (Fig. 4d). Notably, the CDC42-like GTPase 1 RhoU was found to be transcriptionally regulated by STAT3, with its mRNA levels induced on gp130-mediated cytokine stimulation and a significant reduction of its protein level in *Stat3*-null cells^43^, suggesting the possible role of STAT signaling in the transcriptional regulation of the Rho family of GTPases. Particularly, the GRAM domain in myotubularin was shown to be required for the endosomal trafficking of endocytosed EGFR to regulate its activation^44,45^. In support of this, the GRAM domain shares similarities to the GLUE (GRAM-like ubiquitin-binding in Eap45 domain), which binds to ubiquitin that functions to mark endocytosed receptors for lysosomal degradation^46^. In the JAK/STAT signaling pathway, the ligand-receptor internalization and trafficking to the early endosome were reported to be associated with the signaling intensity^47^, suggesting that GRAMD1B may function to promote ligand-receptor decay, causing the de-activation of JAK/STAT signaling.

Another signaling cascade often implicated in breast tumorigenesis is the PI3K/Akt signaling pathway, where it is considered a potential therapeutic target due to its role in tumor initiation and progression^10,48,49^. For example, Ca^2+^-dependent Akt activation was implicated in TRPV4-mediated breast cancer cell migration and metastasis^50^, and Twist-Akt2 signaling axis was shown to be essential in promoting the invasive ability and survival of breast cancer cells^13^. Furthermore, Akt has been found to be required for the formation of membrane ruffles and lamellipodia through interaction with actin filaments and co-localization with the ARP2/3 complex^51,52^. Notably, our data suggested that GRAMD1B acts downstream to PI3K, but upstream/parallel to Akt. The spatial localization of PI3K and its products at the leading edge of motile cells was shown to be crucial for effective cell migration. Specifically, PI3K and its interacting PH domain-containing protein binding partners such as Akt have been reported to translocate to the plasma membrane in response to a chemoattractant stimulus^53,54^. At the leading edge, the membrane translocated Akt interacts with the PI3K products PI(3, 4, 5)P_3_ and PI(3, 4)P_2_, triggering well-coordinated cell movement^53^. Since GRAM domain has been implicated in membrane-coupled lipid/protein-binding, it is conceivable that GRAMD1B may play a role in translocation of Akt and/or de-stabilization of the interaction between Akt and the PI3K products at the leading edge of motile cells. In support of this, in another GRAM domain-containing protein MTMR2, the GRAM domain has been recognized to play a role in PI(3, 5)P_2_ and PI(5)P substrate recognition^55^, thereby highlighting the possible function of GRAM domain in phosphoinositide recognition.

Several reports have indicated a close interaction between the JAK/STAT and PI3K/Akt signaling cascades in promoting metastasis in breast cancer^4,16^. Specifically, co-inhibition of the PI3K/mTOR and JAK2 signaling cascades was found to synergistically reduce breast tumor growth and metastasis, as well as improve overall survival *in vivo*^16^. Therefore, we undertook epistasis analysis to further elucidate the regulatory hierarchy between JAK/STAT and PI3K/Akt signaling cascades on loss of GRAMD1B activity. Interestingly, we detected a dose-dependent increase in p-Akt levels on JAK/STAT inhibition by AG490 (Fig. 6a), however, no alteration in p-STAT3 levels was observed on Akt inhibition by MK-2206 (Fig. 6b), suggesting the inhibitory effect of the JAK/STAT cascade on Akt activity in MDA-MB-231 cells. Importantly, this induction of p-Akt levels was further enhanced in cells transfected with *si*-*Gramd1b* (Fig. 6c), thereby providing new knowledge about GRAMD1B being the central player in regulating the inhibitory effect of JAK/STAT signaling on Akt activity in breast tumorigenesis.

Notably, the JAK/STAT signaling cascade is an important regulatory pathway mediating breast tumor growth and survival^6^. There is evidence supporting a direct correlation between STAT3 activation and increased Cyclin D1 expression in primary breast tumors and breast cancer-derived cell lines^6,56^. Consistently, loss or depletion of STAT3 in breast carcinoma cells has been shown to result in tumor inhibition and induction of apoptosis^57,58^. Several reports also support a pivotal function of the Akt pathway in mediating breast cancer cell proliferation^9,59^, such that its inhibition impedes cell cycle progression and promotes cell death^60^. Since our findings revealed a novel central role of GRAMD1B in negatively regulating the JAK/STAT and Akt pathway, it is conceivable that GRAMD1B may function to inhibit breast cancer cell proliferation and promote cell death. Further analyses exploring the role of GRAMD1B in these cancer hallmarks is necessary to provide a deeper understanding into its exact function in regulating breast tumorigenesis.

Given that the understanding of the mechanisms underlying cell migration can provide crucial insights for the development of anticancer therapeutic agents, the identification of molecules that play an important role in cell motility is therefore imperative in the fight against cancer metastasis. Due to the novelty of GRAMD1B, more studies need to be carried out to further understand the mechanisms by which it regulates breast tumorigenesis through modulating both JAK/STAT and Akt signaling. Nonetheless, our study suggests that GRAMD1B is a key signaling molecule that functions to inhibit cell migration in breast cancer, providing the foundation for its development as a novel biomarker in breast tumors.

## Materials and Methods

### Cell culture, chemicals and transfections

The human MDA-MB-231 breast cancer cell line (ATCC, Rockville, MD) was maintained in RPMI-1640 medium (HyClone^TM^), containing 10% FBS (Gibco) and 1% Penicillin-Streptomycin (Gibco). IL-6 (PeproTech, USA) was used to activate JAK/STAT signaling at working concentrations of 20–60 ng/ml for 6 hours. Cells were treated with the JAK2 inhibitor AG490 (Sigma-Aldrich) and the Akt inhibitor MK-2206 (Selleck, USA) for 24 hours. The Rho inhibitor Rhosin hydrochloride (Tocris Bioscience), Rac1 inhibitor NSC23766 (Santa Cruz Biotechnology) and Cdc42 inhibitor ML141 (Santa Cruz Biotechnology) were reconstituted in DMSO. Small interfering RNA (siRNA) targeting *Gramd1b* (5′GCUCUUAGAGUCCCAACAATT3′; 3′TTCGAGAAUCUCAGGGUUGUU5′) was designed and synthesized by Singapore Advanced Biologics Pte. Ltd. (SABio, Singapore). *si-Gramd1b-2* (Ambion, AM16708) was used to rule out off-target effects of siRNA for *Gramd1b*. Non-targeting siRNA (Ambion) was used as a negative control. Cells were transfected with siRNA using the transfection reagent Lipofectamine 3000 (Invitrogen, USA) as per the manufacturer’s instructions.

### RNA extraction and quantitative real-time PCR (qRT-PCR)

The RNeasy mini kit (Qiagen GmbH, Germany) was used to extract total RNA, which was subsequently converted to cDNA using the Revert Aid First Strand cDNA Synthesis Kit (Thermo Fisher Scientific, USA). The samples were loaded in triplicates in a 96-well plate for each sample set, and quantitative PCR was carried out using the FAST SYBR green cocktail (Applied Biosystems, USA) in the HT7900 FAST Realtime PCR system (Applied Biosystems,USA). Sequence of the primers (IDT technologies) used for this study are listed in Supplementary Table S1.

### Protein isolation and western blot

Protein lysates were extracted using M-PER^TM^ Mammalian Protein Extraction Reagent (Thermo Fisher Scientific, USA) containing Halt^TM^ Protease Inhibitor cocktail and EDTA (Life Technologies, USA). Protein concentration was determined by the Microtiter Bio-Rad Protein Assay solution (Bio-Rad Laboratories, CA, USA). 30 μg of proteins were loaded onto SDS-PAGE gels, and standard western blot analysis was performed. The following antibodies were used: GRAMD1B (Abcam, ab121286), Rac1/RhoA/Cdc42 (Cell Biolabs, Inc., #STA-404), p-STAT3 (Tyr705) (Cell Signaling Technology, #9145), STAT3 (Cell Signaling Technology, #12640), p-JAK2 (Tyr1007/1008) (Cell Signaling Technology, #3771), JAK2 (Cell Signaling Technology, #3230), p-Akt (Ser473) (Cell Signaling Technology, #4060), Akt (Cell Signaling Technology, #4691), p-PI3K p85 (Tyr458)/p55 (Tyr199) (Cell Signaling Technology, #4228), PI3K p85 (Cell Signaling Technology, #4257) and β-actin (Sigma- Aldrich, A2228).

### Immunofluorescence staining

Following 72 hours of transfection with siRNA, cells were fixed using 4% paraformaldehyde, and then permeabilized with 0.1% Triton X-100. For F-actin cytoskeleton staining, the cells were incubated with TRITC-conjugated Phalloidin (Merck Millipore, USA) at 1:500 dilution at room temperature for 1 hour. The slides were imaged under the Confocal Laser Scanning Microscope (Olympus Fluoview FV 1000).

### Transwell migration assay

Following 48 hours of transfection with siRNA, cells were treated with AG490 at 50 μm or MK-2206 at 10 μm for an additional 24 hours. Following the treatment, cells were re-suspended in serum free RPMI-1640 medium and seeded into polycarbonate membrane transwell inserts (Corning Inc., USA). RPMI-1640 medium containing 10% FBS was used as a chemoattractant, and cells were incubated at 37 °C for 18 hours to allow migration. Following incubation, the migrated cells were stained using 0.5% crystal violet and visualized using the Nikon SMZ1500 microscope. The average number of migrated cells per insert was calculated by imaging five different fields.

### Wound healing assay

A linear scratch or wound was made across the confluent monolayer of the 72 hours post transfected cells using a fine 10 μl pipette tip. Three random fields were marked out, and images were subsequently taken at 12 hour intervals to monitor cell migration. The average gap width and average number of migrated cells across the three marked fields were then measured and calculated at each time point.

### Statistical analysis

The GraphPad prism 6 software (GraphPad Prism, USA) was used to carry out statistical analysis. For comparing means between two groups, a two-tailed student T-test was used. A one-way ANOVA was used for tests involving more than two groups. For wound healing assay, the two-way ANOVA statistical test was adopted. Data is represented as means ± SEM, and results are considered statistically significant if *P* < *0.05*.

## Electronic supplementary material

Supplementary Information
