# Supplementary material for: GRAMD1B regulates cell migration in breast cancer cells through JAK/STAT and Akt signaling
Source: Sci Rep. 2018 Jun 22;8:9511. doi: 10.1038/s41598-018-27864-6 (PMC6015000; doi:10.1038/s41598-018-27864-6)

GRAMD1B regulates cell migration in breast cancer cells through JAK/STAT and Akt signaling

Puja Khanna[1], Joan Shuying Lee[1], Amornpun Sereemaspun[2], Haeryun Lee[3], Gyeong Hun Baeg[1,*]

[1]Department of Anatomy, Yong Loo Lin School of Medicine, National University of Singapore, MD10, 4 Medical Drive, Singapore117594

[2]Department of Anatomy, Faculty of Medicine, Chulalongkorn University, Bangkok, Thailand 10330

[3]Department of Life Sciences, Pohang University of Science and Technology, Pohang, South Korea 37673

Correspondence: Dr GH Baeg, E-mail: antbgh@nus.edu.sg

Supplementary Table S1: Primers used for qRT-PCR

Genes	Forward Primer	Reverse Primer
Gramd1b	TGG GGG AGA AGA TTG AGA TG	TGT CCA CGC TGA AGT TGA AG
Rac1	ATG CAG GCC ATC AAG TGT GTG GTG	TTA CAA CAG CAG GCA TTT TCT CTT CC
RhoA	CAG TTC GAG GTG TAT GT	AGA CAA GGC AAC CAG ATT TT
Cdc42	GCC CGT GAC CTG AAG GCT GTC A	TGC TTT TAG TAT GAT GCC GAC ACC A
GAPDH	TGC CAT GGG TGG AAT CAT ATT GG	GAA GGT GAA GGT CGG AGT CAA GG

Supplementary Figure S1: *si-Gramd1b-2* decreases GRAMD1B protein levels in MDA-MB-231 cells.

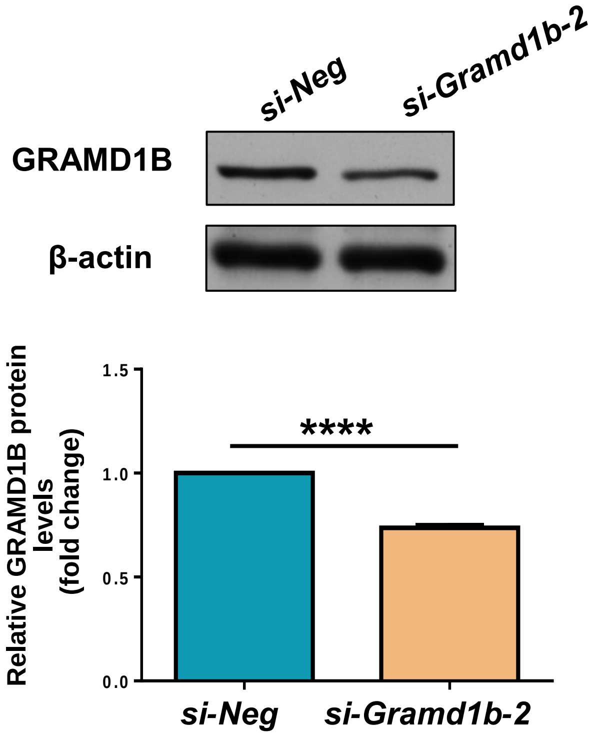

Supplementary Figure S2: *si-Gramd1b-2* induces cell morphology changes in MDA-MB-231 cells.

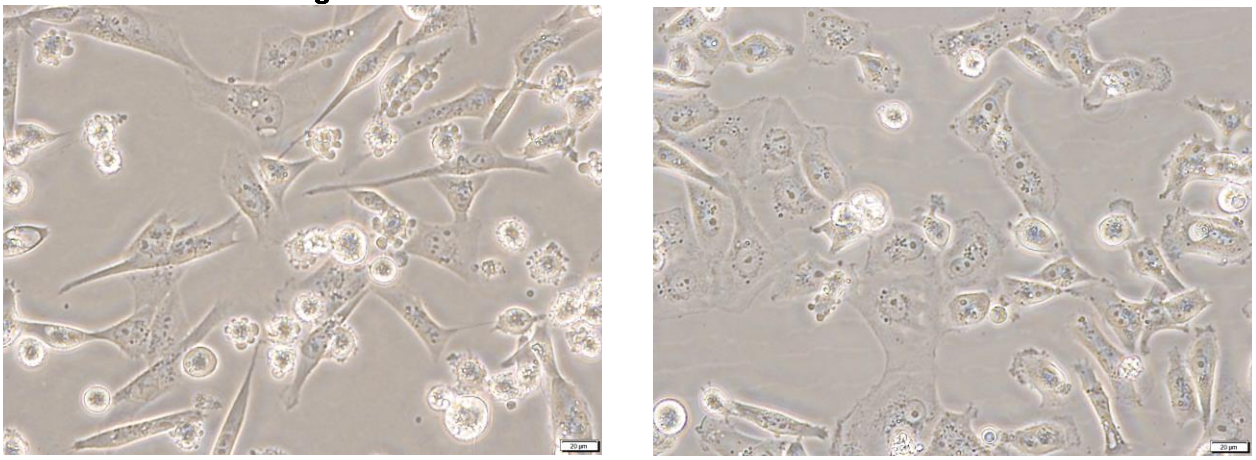

Supplementary Figure S3: GRAMD1B-mediated cell morphology changes is *via* the Rho family of GTPases.

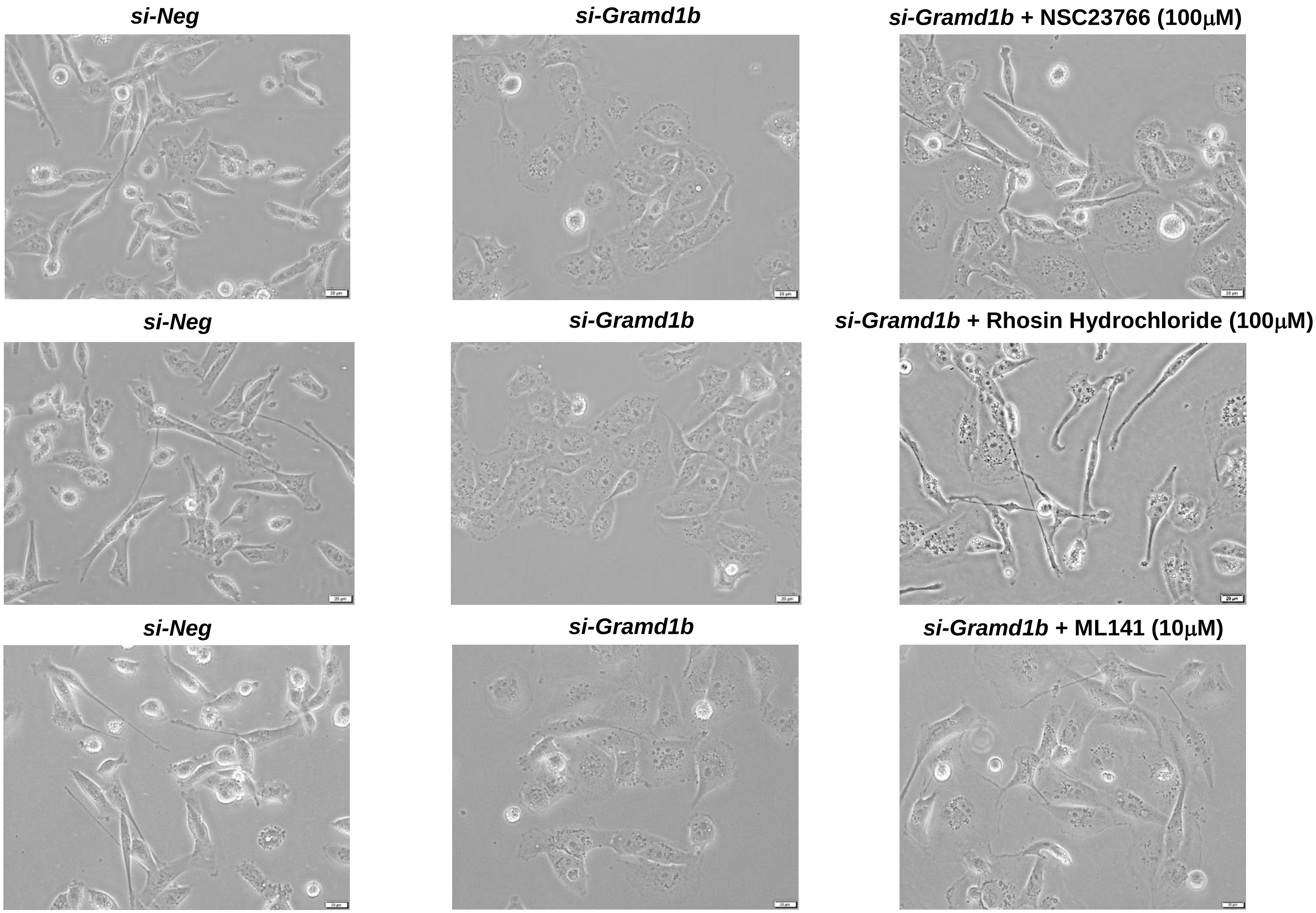

Supplementary Figure S4: No morphology change in MDA-MB-231 cells on MK-2206 treatment.

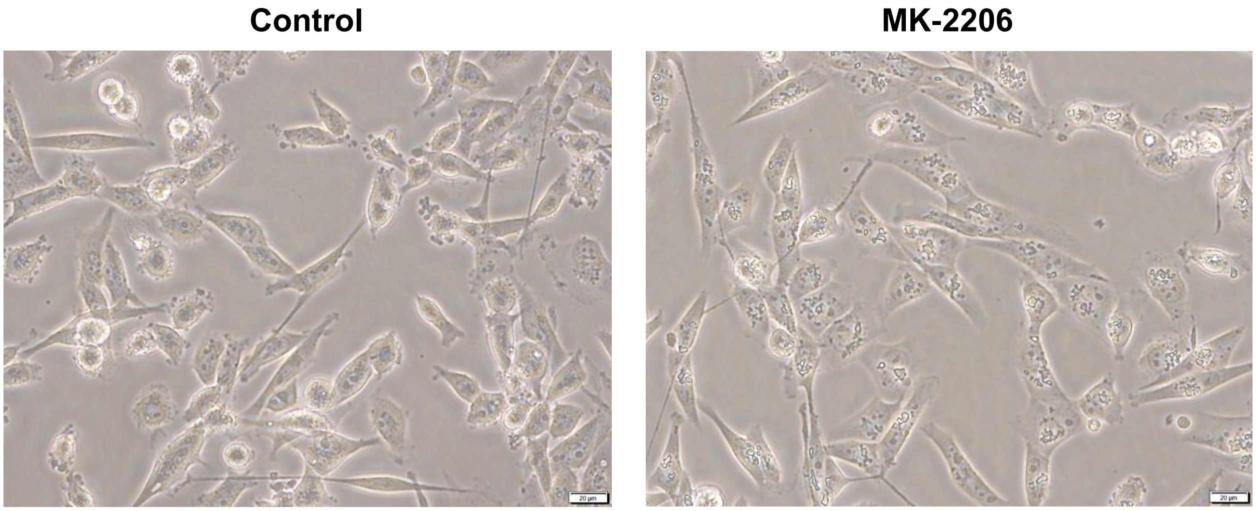

Supplementary Figure S5: Dose-dependent increase in p-Akt levels on AG490 treatment on inhibition of GRAMD1B.

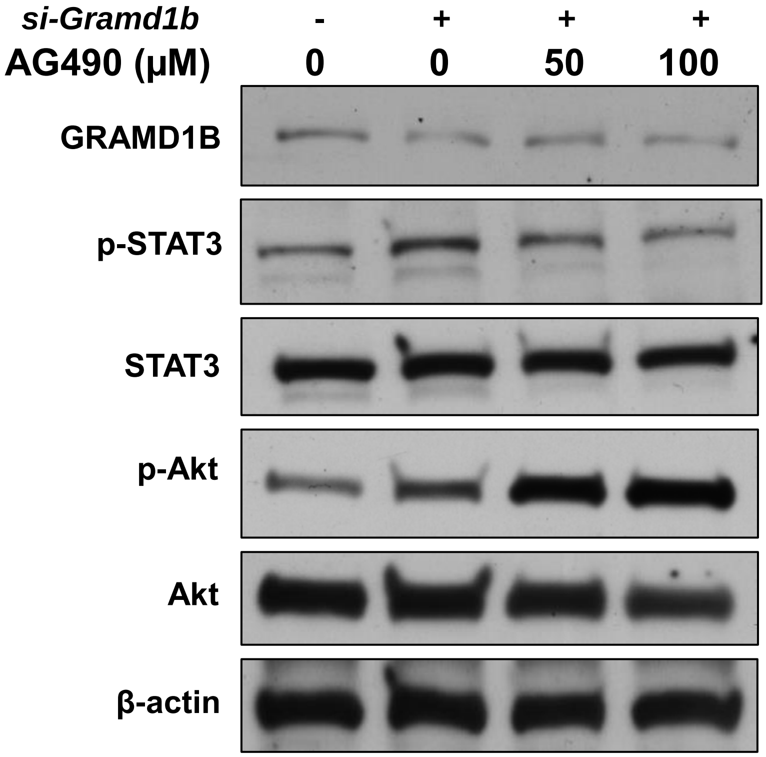

Supplementary Figure S6: Full-length blots of Figure 1a

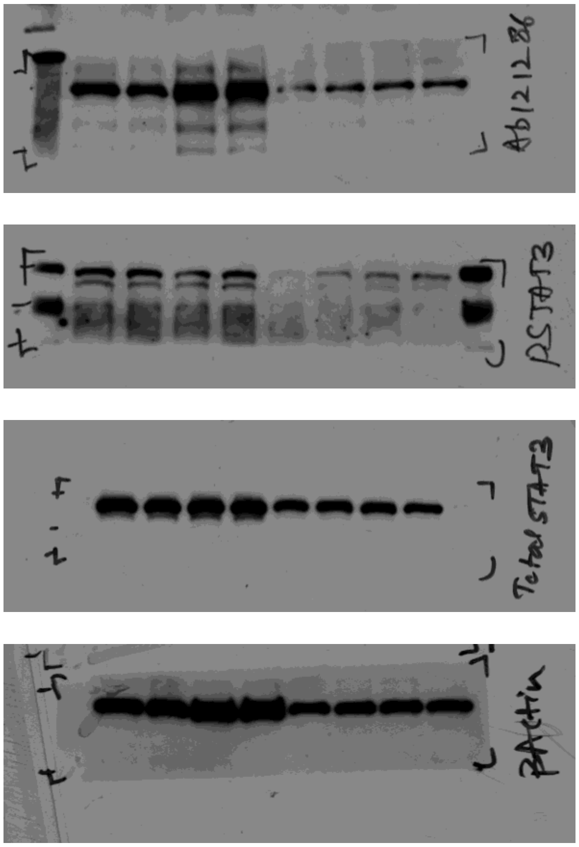

Supplementary Figure S7: Full-length blots of Figure 1b

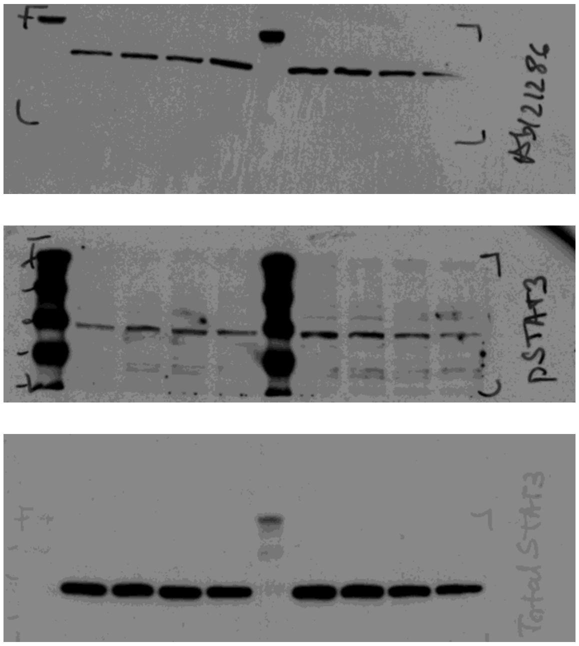

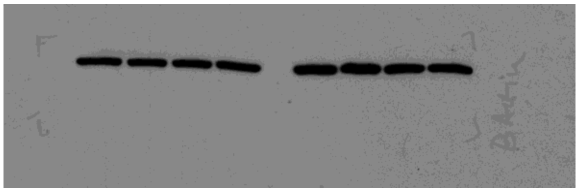

Supplementary Figure S8: Full-length blots of Figure 4a

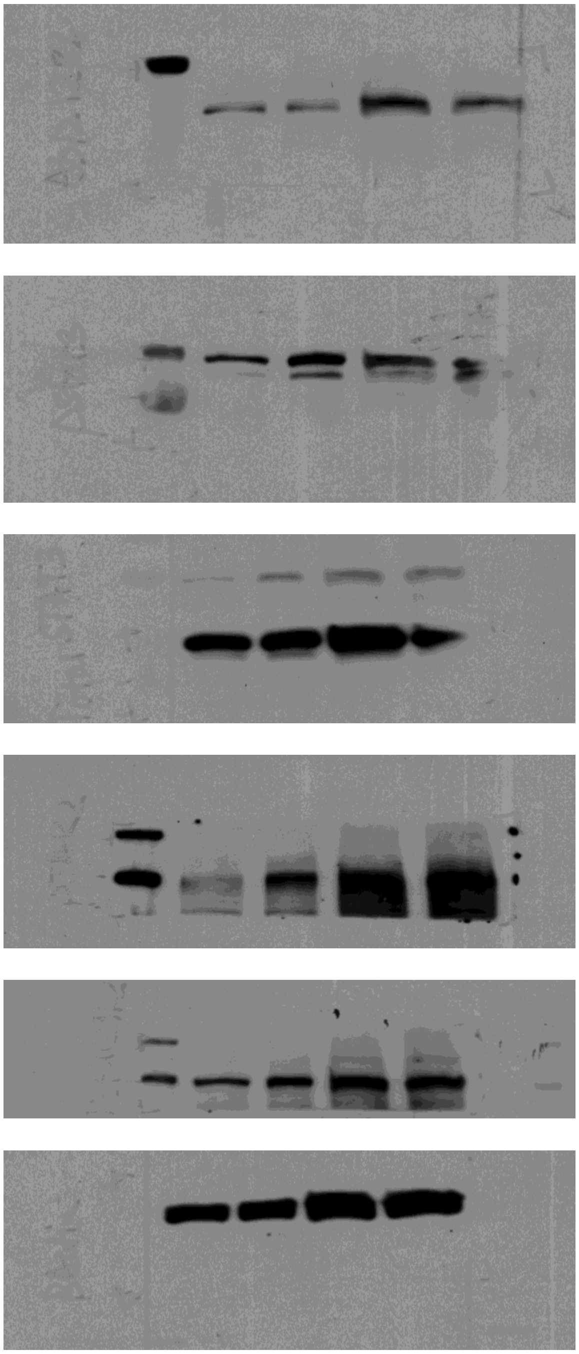

Supplementary Figure S9: Full-length blots of Figure 5a

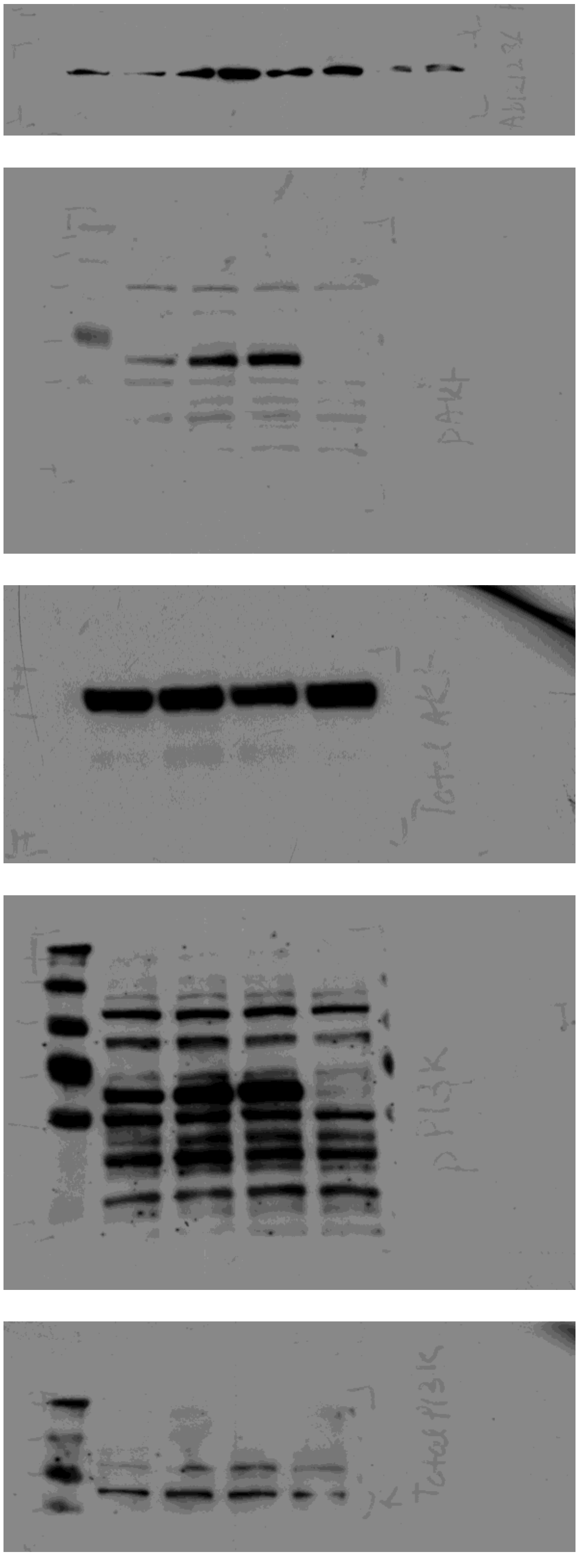

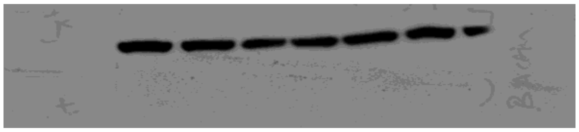

Supplementary Figure S10: Full-length blots of Figure 6a

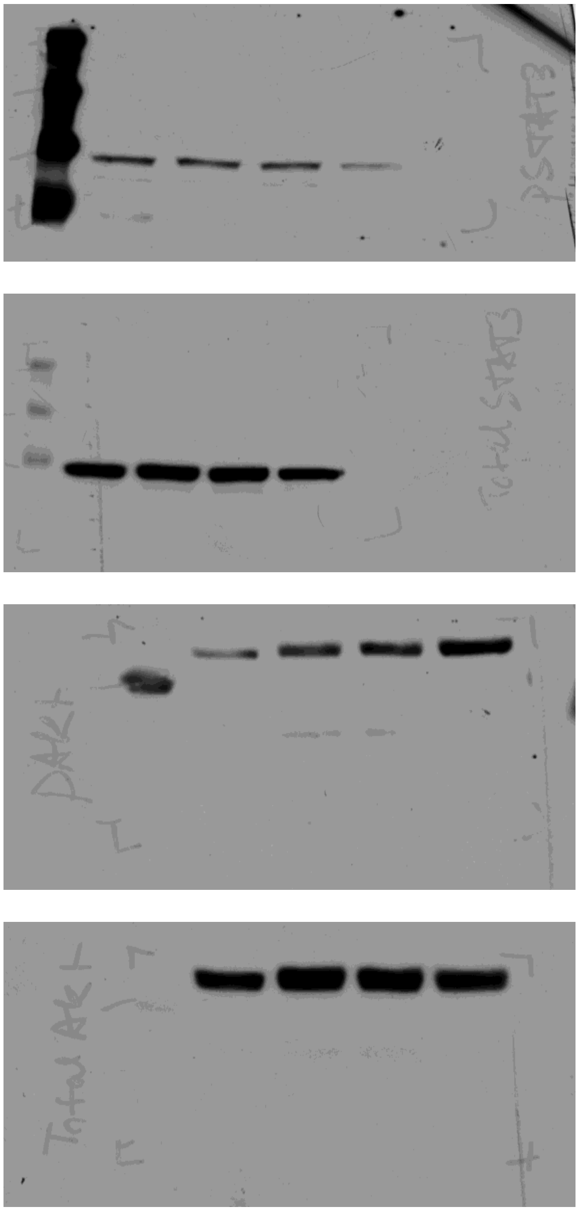

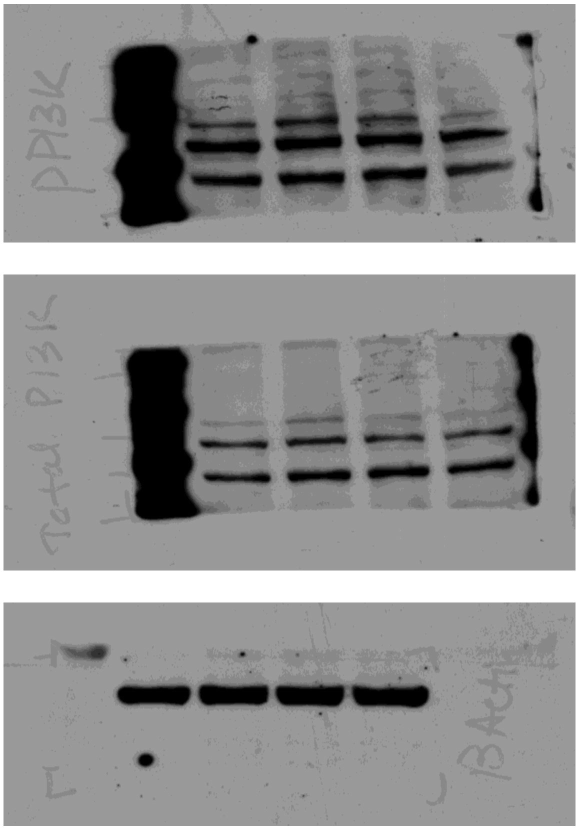

Supplementary Figure S11: Full-length blots of Figure 6b

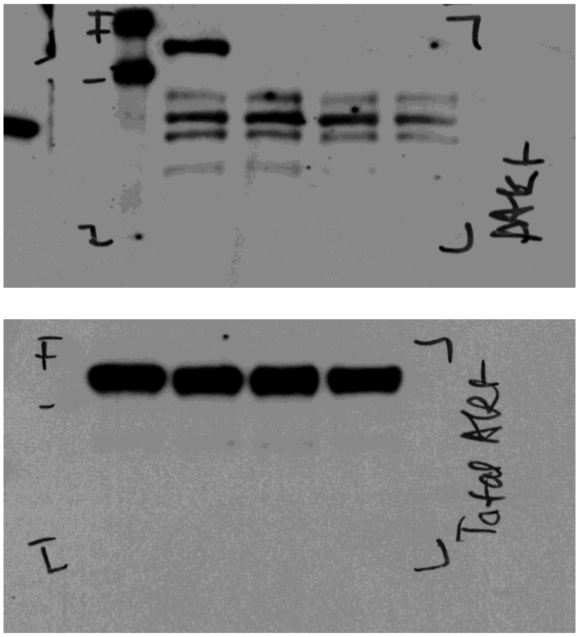

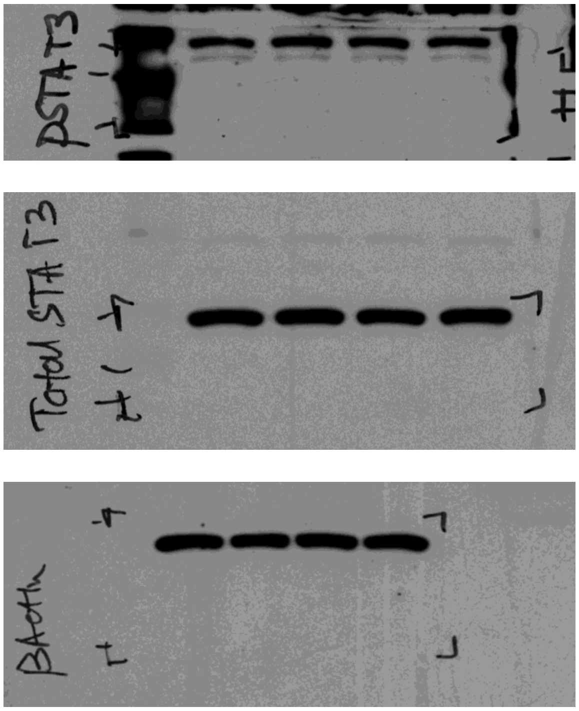

Supplementary Figure S12: Full-length blots of Figure 6c

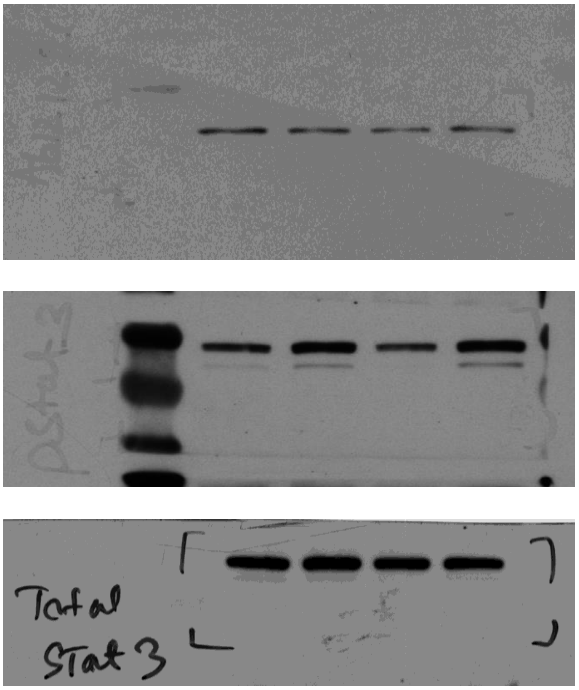

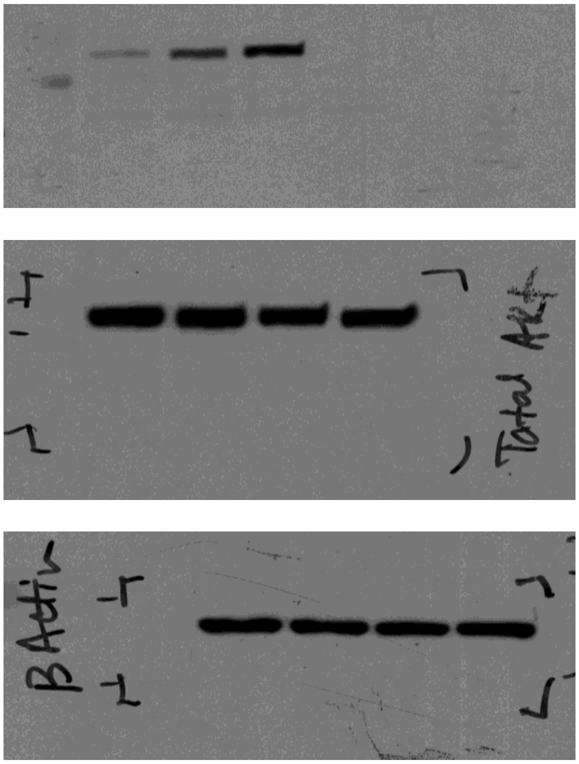

Supplementary Figure S13: Full-length blots of Supplementary Figure S1

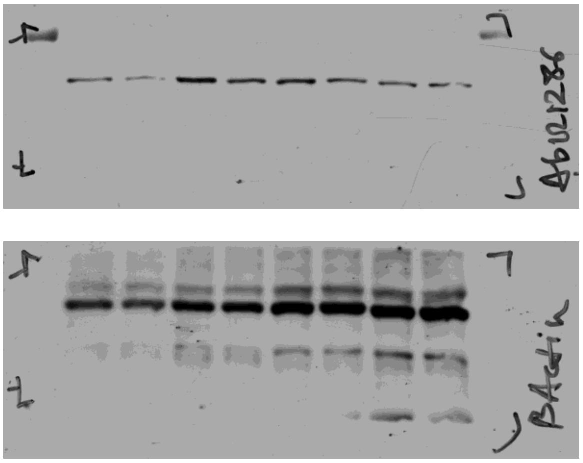

Supplementary Figure S14: Full-length blots of Supplementary Figure S4

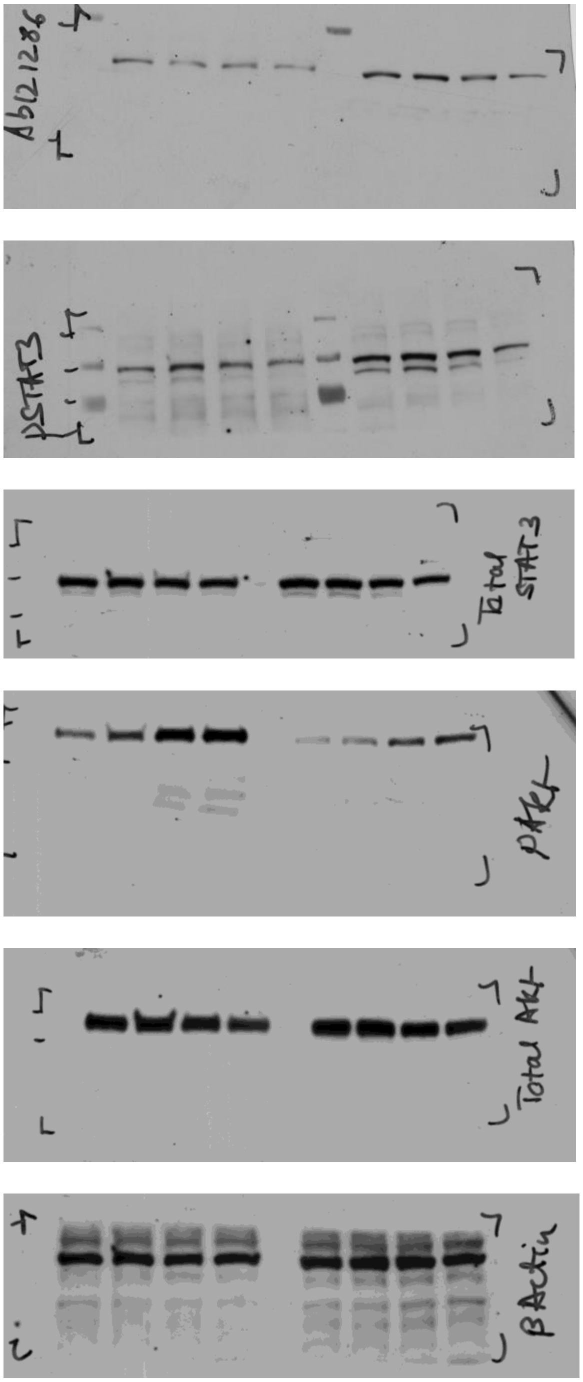

Supplement: Supplementary file 1 — Supplementary Information [file 41598_2018_27864_MOESM1_ESM.pdf]
